# Inhibition of interferon-signalling halts cancer-associated fibroblast-dependent protection of breast cancer cells from chemotherapy

**DOI:** 10.1038/s41416-020-01226-4

**Published:** 2021-01-04

**Authors:** Robyn V. Broad, Stacey J. Jones, Melina C. Teske, Laura M. Wastall, Andrew M. Hanby, James L. Thorne, Thomas A. Hughes

**Affiliations:** 1 School of Medicine, University of Leeds, Leeds, UK; 2 Department of Breast Surgery, Leeds Teaching Hospitals NHS Trust, Leeds, UK; 3 Department of Histopathology, St. James’s University Hospital, Leeds, UK; 4 School of Food Science and Nutrition, University of Leeds, Leeds, UK

**Keywords:** Breast cancer, Cancer therapeutic resistance

## Abstract

**Background:**

Triple negative breast cancers (TNBC) have poor prognoses despite aggressive treatment with cytotoxic chemotherapy. Cancer-associated fibroblasts (CAFs) are prominent in tumour stroma. Our hypothesis was that CAFs modulate chemotherapy sensitivity.

**Methods:**

TNBC cells and breast fibroblasts were cultured; survival after chemotherapeutics was assessed using luciferase or clonogenic assays. Signalling was investigated using transcriptomics, reporters, recombinant proteins and blocking antibodies. Clinical relevance was investigated using immunohistochemistry.

**Results:**

Breast CAFs dose-dependently protected TNBC cell lines MDA-MB-231 and MDA-MB-157, but not MDA-MB-468s, from chemotherapy. CAF-induced protection was associated with interferon (IFN) activation. CAFs were induced to express IFNβ1 by chemotherapy and TNBC co-culture, leading to paracrine activation in cancer cells. Recombinant IFNs were sufficient to protect MDA-MB-231 and MDA-MB-157 but not MDA-MB-468 cells. In TNBC patients, IFNβ1 expression in CAFs correlated with cancer cell expression of MX1, a marker of activated IFN signalling. High expression of IFNβ1 (CAFs) or MX1 (tumour cells) correlated with reduced survival after chemotherapy, especially in claudin-low tumours (which MDA-MB-231 and MDA-MB-157 cells represent). Antibodies that block IFN receptors reduced CAF-dependent chemoprotection.

**Conclusions:**

CAF-induced activation of IFN signalling in claudin-low TNBCs results in chemoresistance. Inhibition of this pathway represents a novel method to improve breast cancer outcomes.

## Background

Breast cancer is the most common malignancy in women and the second most common overall, causing ~600,000 deaths annually worldwide.^1^ Breast cancers are classified clinically into different molecular subtypes,^2^ based mainly on expression of oestrogen receptor, progesterone receptor and HER2, and this classification defines suitable therapeutic options, including agents targeting oestrogen or HER2 function. Triple negative breast cancers (TNBC), which make up ~15% of breast cancers, do not express these markers, and accordingly cytotoxic chemotherapy is the only appropriate systemic therapy.^2^ However, TNBC outcomes are relatively poor despite this aggressive treatment.^3^

The tumour microenvironment has potent and complex influences on breast cancer behaviour.^4^ Cancer-associated fibroblasts (CAFs) are a major cellular component of breast tumour microenvironment,^5^ and have been shown to promote cancer proliferation, invasion and metastases through paracrine signalling pathways, such as secretion of VEGF, FGF2, TGFβ, CXCL12 and IL6, as well as indirectly through modifications to extracellular matrix.^6^ Accordingly, the presence of CAFs is significantly associated with poor outcomes from breast cancer generally,^7^ and in TNBC specifically.^8^ An alternative explanation for this correlation is that CAFs directly influence therapy response, potentially inducing therapy resistance.^9^ The predominant model that links CAFs to therapy response involves CAF-modified extracellular matrix^6,10^ that changes physical tissue properties^11,12^ and therefore drug permeability.^6^ Direct paracrine influences of CAFs on therapeutic response, particularly to cytotoxic chemotherapy in TNBCs, have received relatively little study. Interestingly, some CAF-secreted paracrine mediators of chemotherapy response in TNBC have been identified, for example FGF5 and CXCL12 have been shown to promote resistance to docetaxel in mouse-models^10^ and to paclitaxel in 3D-culture models^13^ respectively. Identification of specific examples of molecular crosstalk between CAFs and TNBC cancer cells, such as these, presents opportunities for inhibition of the interactions and therefore chemo-sensitisation to improve TNBC outcomes.^10^ Our hypothesis was that CAFs directly modulate responses of TNBC cells to cytotoxic chemotherapy, and therefore that the differential responses of tumours may be driven in part by impact of variable CAF activity. We aimed to identify molecules responsible for such cellular crosstalk and to determine whether the signalling could be inhibited to improve chemotherapy responses.

## Methods

### Ethics and patients

Ethical permissions for use of fibroblasts from breast cancer resections, and of archival tissue and associated clinicopathological data from patients was granted by Leeds (East) REC (references: 09/H1326/108, 06/Q1206/180). Patients were diagnosed and treated within Leeds Teaching Hospitals NHS Trust; they were recruited, and informed consent was taken in line with these permissions. For tissue microarrays, patients were diagnosed between 01/01/2008 and 30/03/2013; inclusion criteria were clinically defined as ER, PR, HER2 negative, lack of neoadjuvant therapies, availability of suitable archival (FFPE) tumour tissue, and availability of at least 2 months follow up.

### Reagents

Epirubicin hydrochloride (Sigma; St Louis, USA); recombinant IFNα and IFNγ (Peprotech; Rocky Hill, USA); mouse anti-human IFN Type I R2 antibody (#MMHAR-2; PBL Assay Science; Piscataway, USA); goat anti-human IFN Type II R1 antibody, mouse IgG2A control, goat IgG control (#AF673, #MAB00, #AB-108-C 3; R&D Systems; Minneapolis, USA); rabbit anti-IFNβ1 and anti-claudin-3 antibodies (#PA5-20390, #PA5-16867; ThermoFisher; Waltham, USA); rabbit monoclonal MX1 antibody (#D3W7I; CST; MA, USA).

### Tissue culture

MDA-MB-231, MDA-MB-468 and MDA-MB-157 cells were purchased from ATCC (Manassas, USA) and MDA-MB-231-luc from Cell Biolabs (San Diego, USA). Cells that stably express GFP and firefly luciferase (MDA-MB-231-GFP/luc), or GFP (MDA-MB-468-GFP) were developed by transduction with lentiviruses.^14^ Breast normal fibroblasts (NFs) or CAFs were extracted from breast cancer resections from >1 cm outside tumour margins, or from inside tumour masses, respectively. Fibroblasts were used as primary lines (passage 5–10), or immortalised by viral transduction to over-express hTERT.^15^ CAF-GFP cells were developed by lentiviral transduction.^16^ Cells were cultured (37 °C) in media from Thermofisher (Waltham, USA) with 10% FCS (Sigma; St Louis, USA) and 1% penicillin/streptomycin (final concentrations 100 U/ml and 100 μg/ml). MDA-MB-231, MDA-MB-468 and immortalised fibroblasts were cultured in DMEM. Primary fibroblasts were cultured in DMEM-F12 and 5 μg/ml Fungizone (Sigma; St Louis, USA). MDA-MB-157 cells were cultured in Leibovitz L-15. Cells were cultured in 5% CO_2_/air incubators, except for MDA-MB-157 (sealed flasks in 100% air). Cells were transfected in OptiMEM without serum using Lipofectamine-2000 (Thermofisher; Waltham, USA). ISRE/GAS reporter plasmids and *renilla* plasmid (pRL-TK) were gifts from Andrew Macdonald (Leeds, UK).^17^

### Fluorescence-activated cell sorting (FACS)

FACS was performed on cells from co-cultures or from matched monocultures to allow separate assessment of epithelial and fibroblast components by either colony forming assays or expression analyses. Cells were removed from culture dishes using trypsin/EDTA (Thermofisher; Waltham, USA) and resuspended in RPMI phenol red free media (Thermofisher; Waltham, USA). An Influx 6-way cell sorter (BDBiosciences; San Jose, USA) was used to identify GFP positive cells (488 nm laser), gating on live cells on FSC/SSC. Typically, ~100,000 single cells of either GFP positive, GFP negative or both separately were collected into ice-cold RPMI phenol red free media. Representative flow plots showing three different co-cultures (MDA-MB-231-GFP/luc and CAF; MDA-MB-468-GFP and CAF; MDA-MB-157 and CAF-GFP) are shown (Fig. S1).

### Luciferase assays

Luciferase assays (survival assays—firefly only; reporter assays—dual) were performed using Promega (Madison, USA) reagents and were quantified by plate reader (Mithras-LB940, Berthold; Bad Wildbad, Germany). For survival assays, epirubicin-treated readings were normalised to untreated cultures of the same fibroblast-epithelial proportions to determine relative survival excluding differences in epithelial cell numbers from the differing proportions. For reporter assays, MDA-MB-231 cells were reverse transfected with ISRE or GAS reporters (firefly) and pRL-TK control (*renilla*) for 18 h and were then replated in culture/co-culture with varying proportions of CAFs. Firefly activity was normalised to *renilla*.

### Colony forming (clonogenic) assays

Mono-/co-cultures were established and treated with drugs/controls as described in figure legends. To determine survival in monoculture experiments (for example, recombinant IFN treatments), cells were resuspended in fresh medium lacking drugs or IFNs and replated in technical duplicate 10 cm dishes at 500 cells per plate. Plates were incubated for 14 days undisturbed. Cells were then fixed/stained using Crystal Violet (Sigma; St Louis, USA) in 50% methanol/20% ethanol. Isolated colonies (>40 cells) were counted manually. For experiments involving co-culture, all cultures (including 0% fibroblast/100% epithelial cultures) were sorted to isolate epithelial cells, which were then replated and assessed as above. Reproducibility of colony counts was confirmed: plates representing a range of different colony numbers were counted by two independent scorers; counts were compared; R^2^ correlation coefficient was 0.949, indicating near perfect agreement.

### RNA analyses

For genome-wide transcriptome analyses, 900,000 MDA-MB-231-GFP/luc cells were sorted and RNA was prepared (ReliaPrep RNA minipreps; Promega; Madison, USA). Affymetrix Clariom D microarray (Santa Clara, USA) analyses were performed by Paul Heath (Sheffield University, UK). Affymetrix transcriptome analysis console v3.0 was used to identify significantly differentially expressed genes (fold changes > ±2, ANOVA *p* < 0.05). Genes identified were analysed in ToppGene (https://toppgene.cchmc.org)^18^ using ToppFun. For qPCR of mRNAs, the GoTaq 2-Step RT-qPCR system was used with random primers and GoScript RT (Promega; Madison, USA) following the manufacturer’s protocol. qPCR was performed with GoTaq qPCR master mix and CXR reference dye in technical duplicates or triplicates using QuantStudio5 (ThermoFisher; Waltham, USA) with SYBR settings. Primers were supplied by IDT (Coralville, USA): OAS1 (#74007036), MX1 (#74007039), IFNA2 (#74849839), IFNB1 (#74849836), IFNG (#74849833), ACTB (#74007033). For qPCR of miRNAs, TaqMan miRNA assays were used following the manufacturer’s protocols (ThermoFisher; Waltham, USA). qPCR was performed in technical triplicates using QuantStudio5 with TaqMan settings. Assays were supplied by ThermoFisher (Waltham, USA): miR-155-5p (#4427975), RNU48 (#4427975) Expression was determined relative to ACTB (mRNA) or RNU48 (miRNA) using δδct.^19^

### Tissue microarrays (TMAs) and immunohistochemistry (IHC)

TMAs were constructed as previously.^20^ In brief, suitable tissue areas (tumour with stroma, avoiding poorly cellular areas, necrosis, sclerosis) were identified on haematoxylin/eosin stained slides by histopathologist LMW and three separate 0.6 mm cores of tumour tissue were taken from resection blocks and inserted into grids in recipient wax blocks. Clinicopathological data were collected (Table S1); disease-free survival was defined as time from diagnosis with primary cancer to diagnosis of recurrence, or for those without an event, to last disease-free follow up. IHC was performed broadly as previously.^21^ In brief, 5-μm sections were taken onto SuperFrost plus slides (Menzel-Glaser; Braunschweig, Germany). Sections were dewaxed with xylene, rehydrated with absolute ethanol, and washed in running tap water. Antigens were heat retrieved in 10 mM citric acid buffer (pH 6.0) using a 900 W microwave (10 min, high power). Slides were treated with 0.3% hydrogen peroxide (Thermo Fisher; Waltham, USA) followed by washes in Tris-Buffered Saline (TBS) and incubation in antibody diluent (ThermoFisher; Waltham, USA). Antibodies were diluted in antibody diluent at 1:800 (IFNβ1), 1:500 (claudin-3) or 1:50 (MX1) and were incubated on slides overnight (4 °C). Antibody diluent only was used for no primary controls. Slides were washed with TBS-T (0.1% Tween-20; Sigma; St Louis, USA) and TBS. SignalStain Boost IHC detection Reagent (HRP) and DAB substrate (Cell Signalling Technology; MA, USA) were used according to the manufacturer’s protocols. Slides were counterstained with Mayer’s Haematoxylin, followed by washing in running tap water, Scott’s water and again in running tap water. Slides were mounted under coverslips in DPX (Fluka; Gillingham, UK). Stained sections were digitally scanned using ScanScopeXT (20x) and manually scored using Webscope (Aperio; Vista, CA, USA) with protocols developed by AMH (consultant breast histopathologist). For IFNβ1, intensity of fibroblast staining was scored as 1 (weak), 2 (moderate) or 3 (strong). For MX1, tumour cell cytoplasmic intensity was scored as 0 (negative), 1 (weak), 2 (moderate) or 3 (strong). For claudin-3, staining of tumour cell cytoplasm/membrane was scored as negative or positive. For each antibody, only intensity was scored since proportions of cells staining at these intensities were consistently the vast majority of cells, therefore proportion was not informative. All cores were scored by SJJ, with 10% scored by a second independent scorer (AMH) to allow for statistical analysis of scoring reproducibility. Interscorer concordance was determined using Cohen’s Kappa statistics: 0.725 (MX1), 0.672 (IFNβ1) and 1 (claudin-3), indicating near perfect, excellent or perfect agreement. For MX1 and IFNβ1, scores for individual cases were means of the core scores for that case, and expression was dichotomised into high and low groups using ROC analyses.^22^ For claudin-3, cores for each case were consistently positive or negative, therefore dichotomisation was positive in all or negative in all.

### Statistics

Data were analysed in Prism (v8) (GraphPad; San Diego, USA), except for IHC correlations and survival analyses, which were performed using SPSS (v19) (SPSS; Chicago, USA).

## Results

### Breast CAFs, but not NFs, consistently protect MDA-MB-231 cells from chemotherapy

**Fig. 1:**
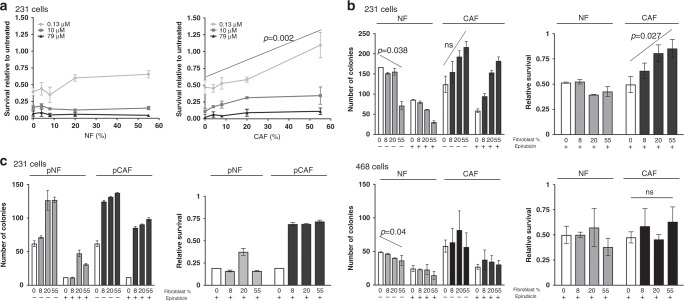
Breast CAFs, but not NFs, protect some TNBC lines from chemotherapy. **a** MDA-MB-231-luc cells were cultured alone (0% fibroblasts) or with increasing proportions of immortalised breast NFs (left panel) or CAFs (right panel). Cultures were treated with three different doses of epirubicin as shown, or with vehicle control for 24 h. Cultures were incubated for a further 48 h in fresh medium, before survival of MDA-MB-231 was assessed using luciferase assays. Data represent survival after epirubicin relative to matched vehicle control cultures, and are means (±SE) of three independent experimental replicates. **b** MDA-MB-231-GFP/luc (top panels) or MDA-MB-468-GFP cells (bottom panels) were cultured alone (0% fibroblasts) or with increasing proportions of immortalised breast NFs or CAFs. Cultures were treated with 10 nM epirubicin or vehicle control for 24 h. Epithelial cells were then collected by FACS and clonogenic survival was determined. Data are presented as colony counts (left panels) or relative survival after epirubicin (colony counts after epirubicin relative to matched untreated cultures; right panels). Data represent means (±SE) of three independent experimental repeats. **c** MDA-MB-231-GFP/luc cells were cultured alone (0% fibroblasts) or with increasing proportions of primary (p) breast NFs or CAFs cultured from a triple negative breast cancer resection. Cultures, cells and data were treated as for part B. Data represent means (±SD) of technical duplicates from one experimental repeat. Statistics: linear regression was carried out for analyses in A and B, with selected significant differences in the overall trend across the fibroblast proportions shown (ns not significant). ANOVA tests were performed in addition; these also demonstrated that CAFs provided significant protection from epirubicin in MDA-MB-231 cells (*p* < 0.01; lowest dose Fig. 1a and Fig. 1b right panel) and not in MDA-MB-468 cells.

Our first aim was to determine whether breast normal fibroblasts (NFs) or CAFs were able to influence sensitivity of TNBC cells to cytotoxic chemotherapy. Initially, we used a short-term co-culture survival assay with luciferase-expressing variants of the TNBC cell line, MDA-MB-231. These were cultured alone (0% fibroblasts) or were co-cultured with either immortalised breast NFs or CAFs in proportions ranging 8–55% fibroblasts. The same total cell number was seeded in each case to achieve comparable overall confluency. Cultures were treated for 24 h with different doses of the anthracycline epirubicin, which is typically used in breast cancer chemotherapy, or with vehicle control; doses approximated to EC50, EC20 and EC10. Relative epithelial cell survival was assessed using luciferase assays (Fig. 1a). As expected, epirubicin reduced epithelial survival dose-dependently (compare *y*-axis positions for different drug doses with 0% fibroblast cultures). The presence of CAFs significantly protected epithelial cells from chemotherapy at the lowest dose of drug (*p* = 0.002) and showed trends for similar protection at both higher doses (*p* = 0.057 and *p* = 0.058), with increasing proportions of CAFs giving increasing protection. Notably, 55% CAFs provided complete protection from the lowest epirubicin dose. NFs, however, showed no significant protection at any dose.

Next, we extended this using an alternative end-point assay, clonogenic survival assays. These assays are more sensitive to lesser degrees of chemotherapy-induced damage, as for cells to count as ‘having survived’ they must be capable of repeated cell divisions. As before, we seeded cultures of TNBC cells either alone or with increasing proportions of breast NFs or CAFs. We used MDA-MB-231 cells, or a second TNBC line, MDA-MB-468, both of which had been transduced to over-express GFP. Cultures were treated with epirubicin or vehicle control for 24 h. Epithelial cells were then separated from fibroblasts by cell sorting on GFP fluorescence and were replated to assess clonogenic potential. Importantly, cultures without fibroblasts were also sorted to allow proper comparison with cells from co-cultures. Data are expressed both as numbers of colonies (Fig. 1b, left), and relative survival after epirubicin (colony numbers after epirubicin treatment relative to matched untreated cultures; Fig. 1b, right). First, it is worth highlighting an unexpected observation in the colony number data in the absence of epirubicin. Although not significant (*p* = 0.087), clonogenicity of MDA-MB-231 cells increased after co-culture with increasing proportions of CAFs, while NFs significantly decreased MDA-MB-231 clonogenicity (*p* = 0.038). CAFs did not confer this increased clonogenicity on MDA-MB-468 cells, although NFs significantly decreased clonogenicity (*p* = 0.04). We concluded that fibroblasts influenced epithelial clonogenicity in a manner unrelated to chemotherapy response. Next, focusing on chemotherapy responses, epirubicin reduced clonogenic survival by ~50% in both MDA-MB-231 and MDA-MB-468 cells in the absence of fibroblasts (Fig. 1b, right; note *y*-axis position of open bars). CAFs, but not NFs, significantly protected MDA-MB-231s from epirubicin in a proportion-dependent manner (*p* = 0.027), with the greatest proportion of CAFs increasing survival to 83% compared to <50% without CAFs. However, CAFs did not protect MDA-MB-468 cells. Moreover, we repeated this experiment with MDA-MB-231 cells and a matched pair of primary breast NFs or CAFs cultured from a triple negative cancer mastectomy specimen (Fig. 1c). We again found that CAFs, but not NFs, provided dramatic protection from chemotherapy.

### CAF-induced chemotherapy protection is associated with upregulation of epithelial IFN signalling

**Fig. 2:**
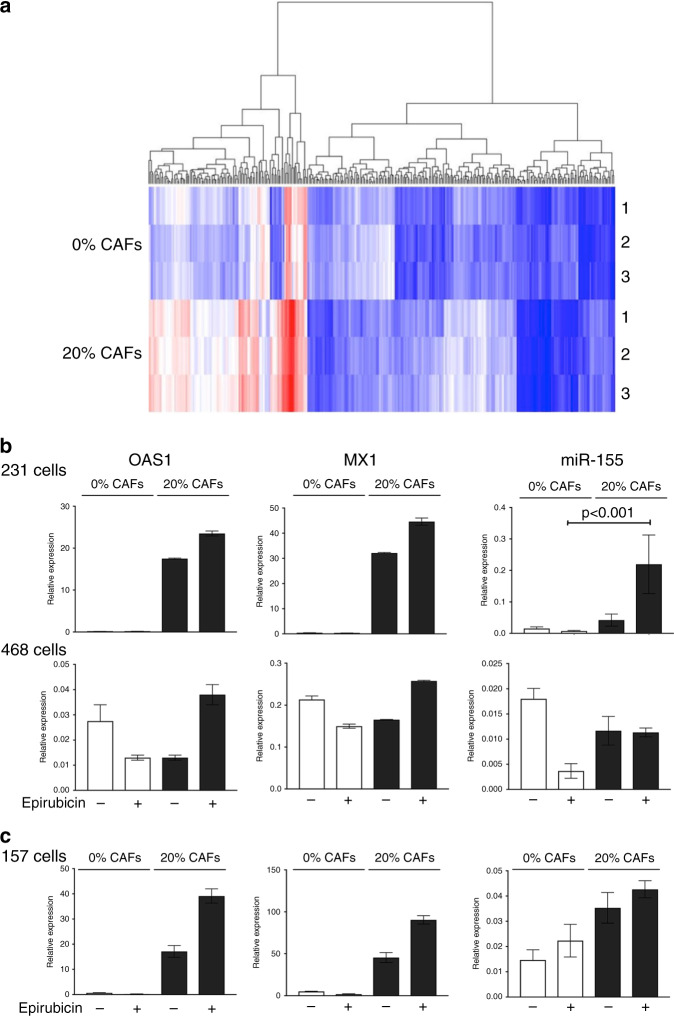
CAFs stimulate IFN signalling in some co-cultured breast epithelial cell lines. **a** MDA-MB-231-GFP/luc cells were cultured alone (0%) or with CAFs (20%) and were treated with 10 nM epirubicin. Epithelial cells were then collected by FACS and RNA was prepared. Three separate biological repeats were performed giving three pairs of samples. Gene expression was assessed using Affymetrix Clariom D microarrays, and comparisons were made between 0% and 20% groups using hierarchical clustering. **b** MDA-MB-231-GFP/luc or MDA-MB-468-GFP cells were cultured on their own (0%) or in combination with CAFs (20%), with or without 10 nM epirubicin. Epithelial cells were collected by FACS and RNA was prepared. Relative expression of interferon response genes OAS1, MX1 and miR-155 was determined using qPCR. **c** MDA-MB-157 cells were cultured on their own (0%) or in combination with CAF-GFP cells (20%), with or without 10 nM epirubicin. Epithelial cells were collected by FACS and RNA was prepared. Relative expression of interferon response genes OAS1, MX1 and miR-155 was determined using qPCR. **b, c** Data represent the mean of technical triplicates (±SD) from one biological experiment, apart from miR-155 analysis in MDA-MB-231 cells, which is from three biological experiments (±SE) and is analysed using two-tailed Mann–Whitney U tests (selected significant difference shown).

Our next aim was to identify gene expression changes induced by CAFs in MDA-MB-231 cells that could be responsible for CAF-induced chemoresistance. MDA-MB-231 cells were cultured on their own (0% fibroblasts) or with 20% immortalised CAFs, were treated with epirubicin, and epithelial cells were collected by cell sorting, exactly as previously. RNA was extracted. This experiment was performed three times over separate weeks to provide robust biological replicates, and gene expression was profiled in the three pairs of samples. Supervised hierarchical clustering was performed to display differences in epithelial expression between the conditions (Fig. 2a), demonstrating that triplicates within each group were similar, and that substantial differences between groups were evident. Paired ANOVA tests were performed to identify significant differences in gene expression of at least 2-fold; 127 genes were significantly more highly expressed in cultures with 20% CAFs, while 57 were more highly expressed in 0% CAF cultures. To define molecular pathways involved, all 184 differentially expressed genes were analysed for pathway enrichment using ToppGene.^18^ The most significantly over-represented pathway was interferon (IFN) signalling, with 12 of the 69 genes annotated as pathway components by the analysis platform included in our input of 184 (*p* = 5.1 × 10^−13^). The 12 differentially expressed IFN-related genes are listed with fold changes in each replicate in Table S2; these included canonical IFN-target genes MX1 and OAS1,^23^ which were upregulated at least 15-fold by CAFs in all three replicates. It was also notable that miR-155, a downstream target of^24^ and positive-feedback regulator of IFN signaling,^25^ was similarly upregulated in MDA-MB-231 cells by CAFs (18.6-fold, ±SD 2.5), as determined by qPCR using the same input RNAs. Overall, these data suggest that IFN signalling was upregulated in MDA-MB-231 cells by CAFs during epirubicin treatment.

Next, we examined whether CAF-dependent upregulation of IFN-related genes differed with or without epirubicin. Therefore, cultures of MDA-MB-231 cells with or without CAFs were established as before, and treated with either epirubicin or with vehicle control, and qPCR was used to assess relative expression of IFN-regulated genes MX1, OAS1 and miR-155 in epithelial cells (Fig. 2b upper panels). We also assessed whether expression of these markers was influenced by CAFs in MDA-MB-468 cells (Fig. 2b lower panels). OAS1, MX1 and miR-155 all demonstrated dramatic CAF-induced upregulation in MDA-MB-231s, with expression potentially further increased by epirubicin treatment (although the effect of epirubicin was not statistically significant). In contrast, MDA-MB-468 showed no CAF-dependent induction, with only minor variation in low basal levels (note the reduced *y*-axis scale). We concluded that these two cell lines showed differential abilities to respond to CAF-dependent upregulation of IFN signalling, which mirrored their abilities to receive CAF-dependent protection from epirubicin (Fig. 1).

MDA-MB-231 and MDA-MB-468 can be classified as claudin-low or claudin-high, respectively.^26,27^ In order to test whether effects of CAFs were potentially related to claudin subtype, the experiment was repeated with a second claudin-low TNBC line, MDA-MB-157. MDA-MB-157s were cultured alone, or with 20% CAFs, and treated with epirubicin or control as before. Epithelial cells were again purified by cell sorting and expression of IFN markers was measured by qPCR (Fig. 2c). Expression in MDA-MB-157 cells was very similar to MDA-MB-231s, with CAF-dependent activation of all three genes. We concluded that CAFs were capable of upregulating IFN signalling in both representatives of claudin-low TNBCs.

### IFNβ1 is upregulated in CAFs after co-culture with MDA-MB-231 cells

**Fig. 3:**
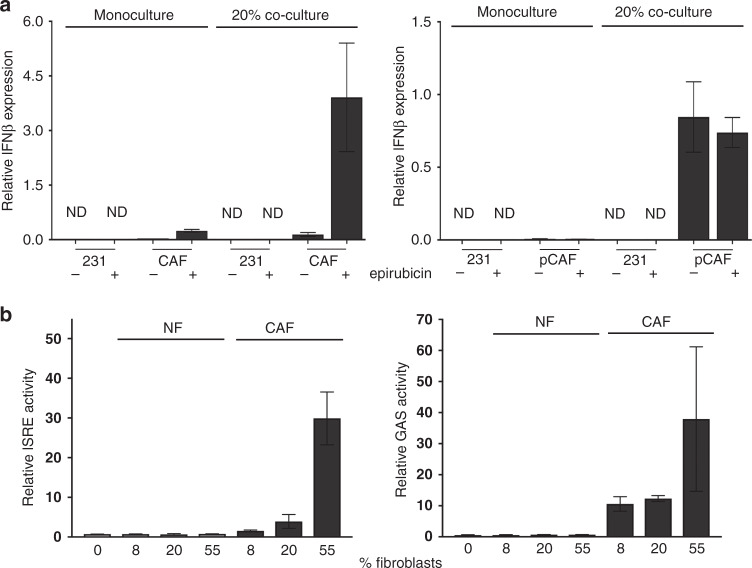
Epithelial:fibroblast crosstalk induces IFNβ1 expression in CAFs and IFN signalling in epithelial cells. **a** MDA-MB-231-GFP/luc cells were cultured alone, breast CAFs were cultured alone, or co-cultures of MDA-MB-231-GFP/luc cells and CAFs were established (80% epithelial cells with 20% fibroblasts: “20%”). CAFs used were either immortalised, left, or primary, right. Cultures were treated with or without 10 nM epirubicin for 24 h. All cultures were processed for cell sorting, allowing separation of CAFs and MDA-MB-231-GFP/luc cells from the co-cultures on the basis of GFP fluorescence in the CAFs. RNA was extracted, and qPCR used to determine relative expression of IFNβ1. Data represent the mean of duplicate culture wells (±SD) for one biological experiment. ND not detected. **b** MDA-MB-231 cells were transfected with ISRE or GAS reporter plasmids driving firefly luciferase expression, and a control plasmid (pRL-TK; HSV thymidine kinase promoter driving renilla luciferase). Transfected MDA-MB-231 cells were then cultured on their own or with different proportions of immortalised NFs or CAFs for 24 h with 10 nM epirubicin. Dual luciferase assays were performed, with firefly readings normalised to renilla readings. Data represent means (±SD) for triplicate wells, for one biological experiment.

Based on these data, our next hypothesis was that CAFs secrete IFNs, stimulating chemoresistance in receptive cells. Therefore, we tested whether we could detect IFN expression using qPCR for IFNα2, IFNβ1 or IFNγ. Monocultures of MDA-MB-231 or CAFs, and co-cultures of MDA-MB-231 and CAFs were established and were treated with epirubicin or vehicle control as before. Co-cultures were separated, using fluorescence-activated cell sorting as previously, to provide purified epithelial and fibroblast samples derived from the co-culture, while the monocultures were also sorted to allow comparison. IFNα2 was undetectable in MDA-MB-231 cells under all conditions, while it was expressed at levels bordering on the limit of detection in CAFs (consequently, there is substantial technical variation); levels in CAFs did not appear to respond to either epirubicin treatment or the presence of epithelial cells (Fig. S2A). Similarly, IFNγ was expressed at very low levels; in CAFs, levels again did not appear to respond to either epirubicin or co-culture, while in MDA-MB-231 cells IFNγ was at least detectable in most samples (Fig. S2B). IFNβ1 was undetectable in MDA-MB-231 cells under all conditions, and in CAFs in monoculture without epirubicin (Fig. 3a, left). However, CAFs were stimulated to express detectable IFNβ1 levels by either epirubicin or co-culture with MDA-MB-231 cells, while epirubicin-treated co-cultures showed dramatic upregulation in CAFs to levels more than three orders of magnitude higher than any detected expression of IFNα2 or IFNγ. Furthermore, we repeated this analysis of IFNβ1 using primary breast CAFs (Fig. 3a, right). Co-culture with MDA-MB-231 cells also stimulated a dramatic increase in IFNβ1 expression in primary CAFs (pCAFs), although epirubicin had little additional effect. We concluded that co-culture with MDA-MB-231 cells stimulated breast CAFs to produce IFNβ1, which may act back on epithelial cells to upregulate IFN signalling (see Fig. 2).

### CAFs, but not NFs, stimulate IFN signalling in co-cultured MDA-MB-231 cells

To confirm that this signalling crosstalk was specific to CAFs, we used IFN activity luciferase reporters in MDA-MB-231 co-cultured with either NFs or CAFs. MDA-MB-231s were transiently transfected with luciferase reporters for activity of ISREs (representing type I IFN signalling) or GAS elements (representing type II IFN signalling), before being placed in monoculture (0% fibroblasts), or co-culture with increasing proportions of either immortalised NFs or CAFs, and treated with epirubicin. Relative luciferase activities were determined (Fig. 3b). NFs did not stimulate either reporter, whereas CAFs induced a proportion-dependent upregulation of both ISRE and GAS activity of up to 30-fold. We concluded that CAFs stimulate IFN signalling in co-cultured MDA-MB-231 cells, but NFs lack this ability.

### Recombinant IFNs are sufficient to protect MDA-MB-231 and MDA-MB-157 cells, but not MDA-MB-468 cells from chemotherapy

**Fig. 4:**
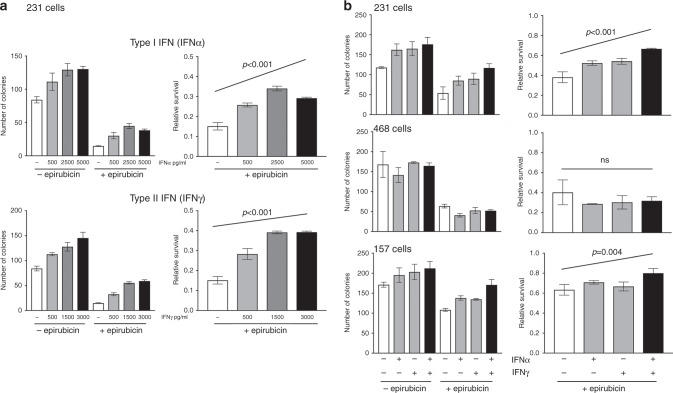
Recombinant IFNs are sufficient to stimulate chemoresistance in MDA-MB-231 and MDA-MB-157 cells. **a** MDA-MB-231-GFP/luc cells were cultured with a range of doses of IFNα or IFNγ for 24 h. Cells were then treated with 10 nM epirubicin, or control, and redosed with IFNs. Clongenic survival was determined as previously. **b** MDA-MB-231-GFP/luc, MDA-468-GFP or MDA-MB-157 cells were cultured with IFNα or IFNγ (2500 pg/ml and 1500 pg/ml, respectively) separately or combination or with appropriate isotype control antibodies for 24 h. Cells were then treated with epirubicin for 24 h (10 nM or, for MDA-MB-157s, 25 nM). Clongenic survival was determined as previously. **a**, **b** Data are shown as either colony counts (left), or survival relative to untreated (right) and represent means (±SE) of either 3 (**a**) or 2 (**b**) independent experiments. ANOVA tests were performed and selected differences are shown (ns not significant).

Our next aim was to determine whether upregulation of IFN signalling in claudin-low TNBC cells was sufficient to induce chemoprotection. To test this, initially we treated MDA-MB-231 cells with a range of doses of a type I IFN (IFNα, which signals through the same pathway as IFNβ1 identified above) or a type II IFN (IFNγ) and determined epirubicin sensitivity using clonogenic survival assays as previously (Fig. 4a). Both IFNs recapitulated effects seen by co-culture with CAFs, in that both provided significant dose-dependent protection from epirubicin (*p* < 0.001). It is interesting to note that both also appeared to increase clonogenicity in the absence of epirubicin (Fig. 4a, left), as was seen previously with CAFs (Fig. 1b, upper left).

Next, we examined effects of recombinant IFNα or IFNγ individually, or in combination, on chemoresponse of MDA-MB-231, MDA-MB-157 or MDA-MB-468 cells (Fig. 4b). IFNα and IFNγ again provided significant chemoprotection to MDA-MB-231 cells, with additive effects when in combination (*p* < 0.001). Similarly, in MDA-MB-157 cells, IFNα and the combination, although not IFNγ alone, provided significant protection (*p* < 0.01). However, there was no significant change in chemoresponse in MDA-MB-468 cells. We concluded that IFNs were sufficient to protect both claudin-low TNBC cell lines, and therefore IFNs were strong candidate mediators of CAF-dependent protection.

### IFNβ1 expression in CAFs and tumour cell expression of MX1 correlate with each other and with poor survival after chemotherapy in TNBC patients

**Fig. 5:**
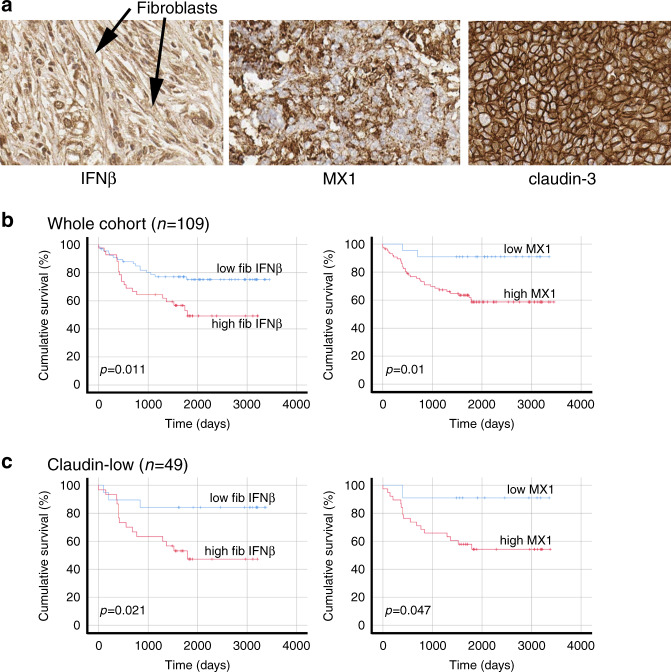
In primary cancers, IFNβ1 in CAFs and MX1 in cancer cells correlate with each other and with poor survival. TMAs of tissue from 109 TNBC resections were assembled and expression of IFNβ1 in fibroblasts, and MX1 and claudin-3 in tumour cells was determined using immunohistochemistry. **a** Representative images of immunohistochemistry, showing tissue scored ‘3’ for IFNβ in fibroblasts (left), ‘3’ for MX1, and ‘positive’ for claudin-3. **b** The cohort was split into groups with high or low expression of IFNβ1 in fibroblasts (left) or MX1 in tumour cells (right) using ROC analyses. Cumulative disease-free survival in the groups was compared using Kaplan-Meier analyses and log rank tests. **c** The cohort was split into claudin-low or claudin-high groups, based on expression levels of claudin-3 (positive or negative). The claudin-low group (*n* = 49) were analysed as in **b**.

Next, we determined whether expression of the molecules we have implicated in chemoresistance correlated with survival after chemotherapy in patients. We collected 109 TNBC resection samples, supported by clinicopathological data including length of disease-free survival. We constructed tissue microarrays containing triplicate cores of cancer tissue and assessed expression of IFNβ1 in fibroblasts and MX1 as a marker of active IFN signalling in tumour cells using immunohistochemistry. We also determined whether individual cases could be classified as claudin-low, using immunohistochemistry for claudin-3. Representative images are shown in Fig. 5a. High expression of IFNβ1 in fibroblasts was weakly, but significantly, positively associated with high MX1 expression in the tumour cells (Spearman’s correlation r = 0.210; *p* = 0.028), suggesting that signalling between the cell types was active. High expression of IFNβ1 in fibroblasts, and MX1 in tumour cells were each significantly associated with poorer disease-free survival (by means of almost 800 days; *p* < 0.02 for both; Fig. 5b).

The cohort was also divided into claudin-low (claudin-3 negative; *n* = 49) and claudin-high (claudin-3 positive; *n* = 60) subgroups. Interestingly, IFNβ1 expression was significantly different between these two groups, with claudin-low tumours expressing overall higher levels (mean scores 2.6 [SD 0.59] vs 2.0 [SD 0.52]; Mann–Whitney *p* = 0.001). The correlation between fibroblast IFNβ1 and tumour cell MX1 was strengthened in the claudin-low group (r = 0.375; *p* = 0.008) while it was lost in the claudin-high cohort (r = 0.113; *p* = 0.389), mirroring our tissue culture observations that claudin-low cancers are most receptive to CAF-induced IFN signalling. Similarly, correlations between survival and expression of each of IFNβ1 in fibroblasts and MX1 in tumour cells were maintained in claudin-low cases (*p* < 0.05; Fig. 5c) but lost in claudin-highs (Fig. S3). We also carried out multivariate analyses to assess whether IFNβ1 in fibroblasts and MX1 in tumour cells provided prognostic insights that were independent of the standard prognostic factors, lymph node status and tumour grade. In the whole cohort, lymph node status and fibroblast IFNβ expression were significant independent predictors of disease-free survival (with hazard ratios of 2.24 [*p* = 0.007] and 2.99 [*p* = 0.001], respectively). In the claudin-low subgroup, both these factors remained significant, with increased hazard ratios (3.77 [*p* = 0.034] and 3.52 [*p* = 0.015], respectively). In the claudin-high subgroup, none of the factors were significantly associated with outcome, although lymph node status demonstrated borderline significance (hazard ratio 2.52 [*p* = 0.052]). We concluded that correlations between IFNβ1 and MX1 and survival in breast cancer patients exactly reflect relationships identified in vitro, with CAF-induced IFN activity correlating with chemoresistance and consequently poor survival, specifically within claudin-low cancers.

### IFN-blocking antibodies inhibit CAF-dependent chemoprotection of cancer cells

**Fig. 6:**
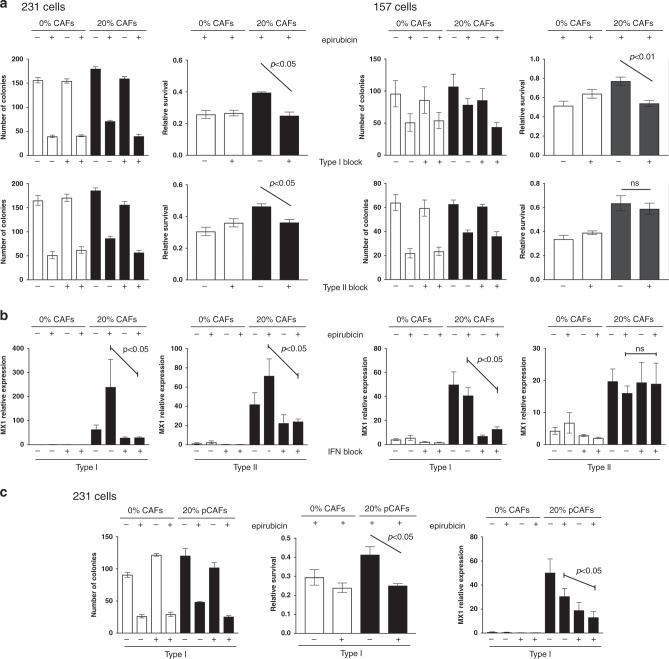
Blocking antibodies inhibit CAF-induced chemoprotection. **a,** **b** MDA-MB-231-GFP/luc (left) or MDA-MB-157 (right) cells were cultured alone, or with breast CAFs or CAF-GFP cells, respectively. Cultures were treated with type I (1 μg/ml) or type II (5 μg/ml) interferon-signalling blocking antibodies or appropriate isotype controls for 24 h. Cultures were then treated with 10 nM (MDA-MB-231 cells) or 25 nM (MDA-MB-157 cells) epirubicin or vehicle control, and were redosed with antibodies for a further 24 h. Epithelial cells were then collected by FACS. **a** Clonogenic survival was determined. Data are presented as colony counts or relative survival after epirubicin (colony counts relative to untreated). **b** Relative expression of the marker of IFN-signalling activity MX1 was determined. **c** MDA-MB-231-GFP/luc cells were cultured alone, or with primary breast CAFs and were treated with antibodies and epirubicin/control exactly as above. Clonogenic survival was determined (left): data are presented as colony counts or relative survival after epirubicin (colony counts relative to untreated). Relative expression of the marker of IFN-signalling activity MX1 was also determined (right). **a**, **b,** **c** Data represent means (±SE) of three independent experimental repeats. Two-tailed Mann–Whitney U tests were carried out and selected differences are shown (ns not significant).

Having defined molecular mechanisms involved in CAF-dependent chemoprotection, we wished to test whether these mechanisms could be inhibited, thereby potentially allowing chemo-sensitisation. We selected antibodies that have previously been used for blocking either type I or type II IFN receptors.^28,29^ MDA-MB-231 or MDA-MB-157 cells were again cultured with or without CAFs, and cultures were treated with type I or type II blocking antibodies, or appropriate isotype controls. Cells were then treated with epirubicin or vehicle control, and epithelial survival was determined (Fig. 6). MDA-MB-231 cells were significantly protected from epirubicin by CAFs as previously (*p* < 0.05), and this protection was significantly reduced by either blocking antibody (*p* < 0.05; Fig. 6a, left); notably, CAF-dependent protection was completely inhibited with the type I antibody. MDA-MB-157 cells behaved similarly, although only the type I antibody significantly inhibited protection (*p* < 0.01; Fig. 6a, right). Importantly, we also used qPCR to assess MX1 expression as a marker of IFN-signalling activity (Fig. 6b). We again confirmed CAF-dependent upregulation of MX1 (as previously in Fig. 2), but also established that blocking antibodies successfully inhibited this upregulation in every case in which blocking antibodies also halted CAF-dependent protection (*p* < 0.05), but not in the one example where blocking antibody was ineffective (type II antibody, MDA-MB-157). We also repeated the experiment using primary breast CAFs, MDA-MB-231 cells and the type I blocking antibody and demonstrated the same significant ability to block CAF-dependent protection (*p* < 0.05; Fig. 6c). We concluded that CAF-dependent protection of breast cancer lines required induction of IFN signalling, and, excitingly, that this can be inhibited in order to chemo-sensitise cancer cells. However, it should be noted that the data shown represent only one selected dose of epirubicin, and we have not assessed the formal impact of CAFs and IFN-blocking antibodies on a chemotherapy dose-response.

## Discussion

Outcomes for TNBC remain relatively poor,^3^ and much research is aimed towards identifying novel therapeutic targets and agents in this breast cancer subtype.^30^ An alternative approach is to use the therapies already available more effectively, a strategy that drives the need to understand mechanisms underpinning chemotherapy resistance better.^31^ In this work, we identify a resistance mechanism that acts in a subset of TNBC using in vitro models, and we confirm its relevance using a patient cohort.

We show that breast CAFs protect claudin-low TNBCs from chemotherapy through secretion of IFNβ1 leading to paracrine activation of IFN signalling in the cancer cells, as denoted by upregulation of MX1. Our data tie together previous reports showing that CAFs are associated with poor outcomes in TNBC patients,^8^ that breast CAFs can secrete IFNβ thereby activating IFN signalling and influencing behaviour of breast cancer cells in vitro,^32,33^ and that expression of MX1 in breast cancer cells is significantly associated with poor outcomes in patients.^34^ Critically, we define the functional impact of this signalling on cancer cells in terms of chemotherapy resistance (Figs. 1 and 4), and indeed chemotherapy-treatment itself contributes to induction of full paracrine activity (Figs. 2 and 3;^33^). This is in contrast to much of the literature on CAFs that defines their influence in terms of inducing proliferation, invasion or metastases, and therefore poor outcomes.^5,6^ This distinction is important clinically, since potential inhibition of CAF-induced chemoresistance could be useful therapeutically,^10,35^ whereas potential inhibition of CAF-induced invasion/metastases is more problematic as these processes are thought to occur prior to breast cancer diagnoses. A further previous study also identified chemoresistance-associated crosstalk between fibroblasts and claudin-low breast cancer cells,^36^ although there are key mechanistic differences with our work. Boelens et al. demonstrated that immortalised lung fibroblasts protected both MDA-MB-231 and MDA-MB-157 cells from chemotherapy via activation of NOTCH3 and STAT1, a key IFN-signalling intermediate, and this was associated with upregulation of IFN response genes OAS1 and MX1.^36^ However, by marked contrast with our work, the authors determined that cellular crosstalk was mediated by RNA transfer via exosomes, independently of IFN or IFN receptors. We demonstrate by use of IFN-blocking antibodies that this action of breast CAFs is entirely dependent on canonical paracrine IFN signalling (Fig. 6), and we conclude that different fibroblasts signal using different mechanisms. We also suggest our use of both primary and immortal breast fibroblasts may be most relevant.

A key discussion point is how these insights could be used to improve cancer outcomes. It is conceivable that alternative treatments could be used for patients whose TNBC tumours display the characteristics identified here as associated with potential anthracycline resistance, namely active IFN signalling between CAFs and claudin-low tumour cells (CAF IFNβ expression/cancer cell MX1 expression; Fig. 5). However, anthracyclines, often combined with taxanes, are the mainstay of TNBC chemotherapy and comprehensive alternatives are not available, although PARP inhibitors and immune check-point inhibitors show potential in some settings.^37^ A more practicable option may be to inhibit crosstalk between CAFs and tumour cells in order to sensitise cancer cells to the existing chemotherapy agents; we present proof of this principle in Fig. 6. We have used receptor-blocking antibodies experimentally since these not only inhibit the pathway required but also specifically target the paracrine aspect of the signalling we wished to prove. This approach also has clinical potential, since a humanised type I receptor-blocking antibody, Anifrolumab, is available and has undergone clinical investigation in lupus.^38^ In addition, ruxolitinib is a small molecule inhibitor of the JAK1/2 kinases, which are IFN-signalling intermediates, that has already been trialled at phase 2 in combination with cytotoxic chemotherapy in metastatic breast cancer.^39^ We conclude that available agents may present opportunities for assessment of therapeutic chemo-sensitisation in the relatively near-term.

The claudin-low breast cancer subtype was identified more than a decade ago;^40^ however, claudin expression levels are not assessed in routine breast cancer management since they have not been found to be useful in directing treatment choices to improve outcomes.^41^ Robust clinical identification of the claudin-low phenotype would be required for therapeutic interventions targeting the CAF-dependent chemoresistance we describe. It remains unclear why claudin-high tumours fail to be protected from chemotherapy by CAFs. Our data from patient samples suggest that claudin-high tumours have lower CAF IFNβ1 levels, therefore, one explanation is less IFNβ1 to activate the pathway. However, we also show that the IFNβ1 present does not correlate with IFN-target gene expression in claudin-high cancer cells, and that the claudin-high cell line, MDA-MB-468, fails to respond to either CAFs or recombinant IFNs in vitro, pointing to a more profound signalling defect. MDA-MB-468 cells have previously been shown to activate signalling downstream of the Type II ligand IFNγ,^42^ although other published data for Type I signalling, as stimulated by IFNβ1, are lacking. Therefore, candidate defects include variation in expression/function of the Type I receptor, IFNAR1, which is known to vary in breast cancer and this variation correlates with prognosis,^43^ or aberrant expression of interferon regulatory factors (IRFs), which act to modulate the range and extent of IFN-target gene activation, and are also known to be deregulated in breast cancer.^44^

## Supplementary information

S material

## Data Availability

Āll data are available either within the manuscript and supplementary material, or directly from the corresponding author.
