# Supplementary material for: Inhibition of interferon-signalling halts cancer-associated fibroblast-dependent protection of breast cancer cells from chemotherapy
Source: Br J Cancer. 2021 Jan 4;124(6):1110–20. doi: 10.1038/s41416-020-01226-4 (PMC7960738; doi:10.1038/s41416-020-01226-4)
Supplement: Supplementary file 1 — S material [file 41416_2020_1226_MOESM1_ESM.pdf]

**Supplementary material**

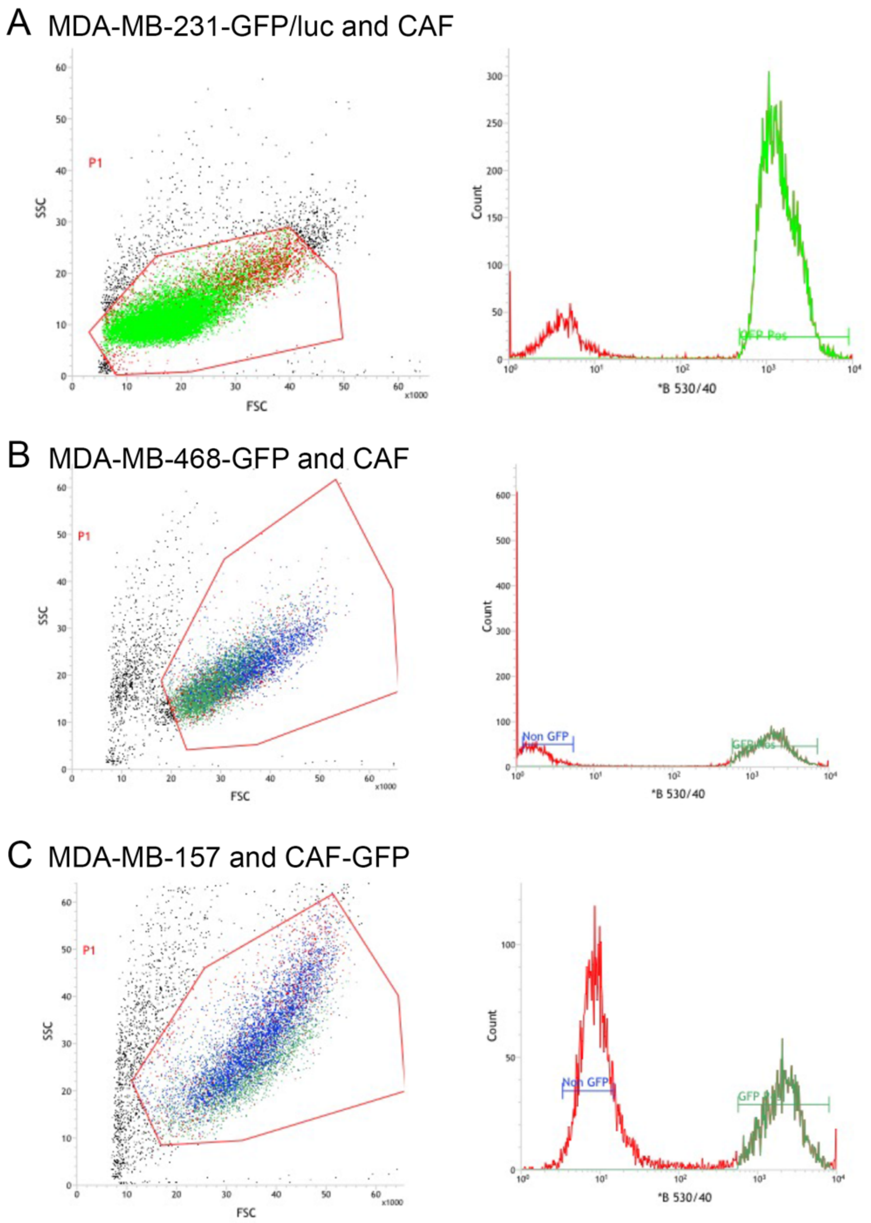


**Figure S1. Epithelial cells and fibroblasts in co-cultures were effectively differentiated on the basis of GFP expression.** Representative flow-cytometry plots are shown for co-cultures of: (A) MDA-MB-231-GFP/luc cells and CAFs (GFP negative); (B) MDA-MB-468-GFP cells and CAFs (GFP negative); and, (C) MDA-MB-157 cells and CAF-GFP cells. In each case, viable cells were first gated (FSC vs SSC, left panel), followed by gating on GFP expression (right plots show a histogram of B540/30 [GFP] intensity against cell number, with GFP negative and positive populations indicated).

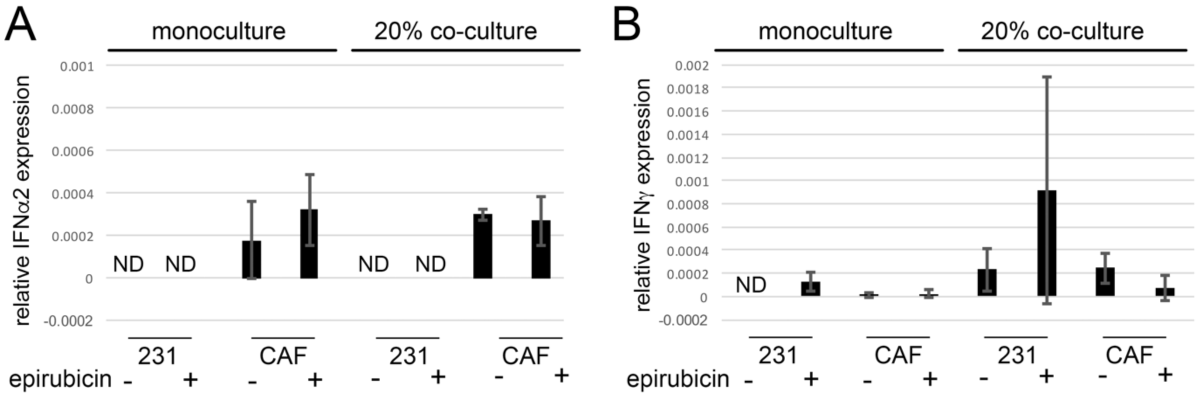


**Figure S2. IFNα2 and IFNγ were either not detected, or were expressed at very low levels, and were not induced by epirubicin or co-culture.** MDA-MB-231-GFP/luc cells were cultured alone, immortalised breast CAFs were cultured alone, or co-cultures of MDA-MB-231-GFP/luc cells and CAFs were established (80% epithelial cells with 20% fibroblasts: "20%"). Cultures were treated with or without 10nM epirubicin for 24h. All cultures were processed for cell sorting, allowing separation of CAFs and MDA-MB-231-GFP/luc cells from the co-cultures on the basis of GFP fluorescence in the CAFs. RNA was extracted, and qPCR used to determine relative expression of IFNα2 (A) and IFNγ (B). Data represent the means of technical duplicates (+/-SD) for 1 biological experiment. ND: not detected. Note the y-axis scale in comparison to Fig 3A; expression levels in Fig S2 are orders of magnitude lower.

Claudin-high (n=60)

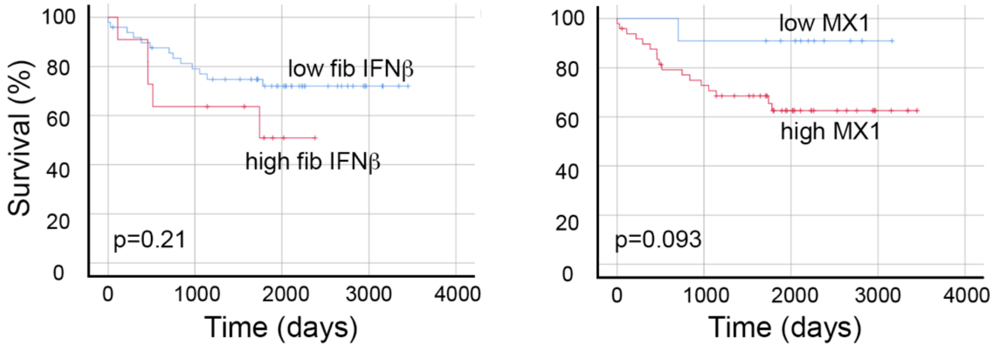


**Figure S3. IFNβ1 in CAFs and MX1 in cancer cells do not correlate significantly with survival in claudin-high TNBCs.** TMAs of tissue from 109 TNBC resections were assembled and expression of IFNβ1 in fibroblasts, and MX1 and claudin-3 in tumour cells was determined using immunohistochemistry. The claudin-high group was identified, based on positive of claudin-3 (n=60). The cohort was split into groups with high or low expression of IFNβ1 in fibroblasts (left) or MX1 in tumour cells (right) using ROC analyses. Cumulative disease-free survival in the groups was compared using Kaplan-Meier analyses and log rank tests.

| **Characteristic** | **n=109 (100%)** |
| --- | --- |
| Age: 57.7 (33-90) years | |
| Follow-up (months): mean 65.6 (range: 2.1-123.2) | |
| **Tumour histopathology:** | |
| Ductal NST | 96 (88) |
| Lobular | 1 (1) |
| Mixed | 3 (3) |
| Metaplastic | 2 (2) |
| Adenoid cystic | 2 (2) |
| Neuroendocrine | 1 (1) |
| Clear cell | 1 (1) |
| Medullary | 2 (2) |
| Inflammatory | 1 (1) |
| **Grade:** | |
| 1 | 1 (1) |
| 2 | 14 (13) |
| 3 | 91 (83) |
| unknown | 3 (3) |
| **Chemotherapy:** | |
| Anthracycline-based | |
| - without taxanes | 56 (51) |
| - with taxanes | 42 (39) |
| - with others | 5 (5) |
| Non anthracycline-based | 6 (6) |

**Table S1. Clinico-pathological features of the patients and their tumours included in the study.**

| IFN-related gene | Fold change 1 | Fold change 2 | Fold change 3 |
| --- | --- | --- | --- |
| IRF9 | 8.41 | 5.17 | 3.42 |
| STAT2 | 4.3 | 4.6 | 2.06 |
| OAS1 | 36.36 | 21.93 | 37.03 |
| OAS2 | 15.52 | 4.71 | 5.95 |
| MX1 | 46.38 | 32.23 | 15.66 |
| MX2 | 18.43 | 13.66 | 10.79 |
| IFI6 | 11.91 | 13.35 | 3.25 |
| IFI44L | 12.04 | 8.37 | 11.83 |
| IFI27 | 12.92 | 7.87 | 6.2 |
| IFITM1 | 5.56 | 4.71 | 3.09 |
| USP18 | 7.13 | 9.15 | 5.15 |
| XAF1 | 7.83 | 3.93 | 3.74 |

**Table S2. CAFs stimulate up-regulation of IFN-related genes in some co-cultured MDA-MB-231-GFP/luc cells.** MDA-MB-231-GFP/luc cells were cultured alone or with CAFs and were treated with 10nM epirubicin. Epithelial cells were then collected by FACS and RNA was prepared. Three separate biological repeats were performed giving three pairs of samples. Gene expression was assessed using Affymetrix Clariom D microarrays, and comparisons were made between 0% and 20% groups. Fold changes are shown from the three replicate comparisons for genes within the IFN-signaling pathway.
